# Preclinical study of mouse pluripotent parthenogenetic embryonic stem cell derivatives for the construction of tissue-engineered skin equivalent

**DOI:** 10.1186/s13287-016-0407-z

**Published:** 2016-10-22

**Authors:** Yang Rao, Jihong Cui, Lu Yin, Wei Liu, Wenguang Liu, Mei Sun, Xingrong Yan, Ling Wang, Fulin Chen

**Affiliations:** 1 Laboratory of Tissue Engineering, College of Life Sciences, Northwest University, Taibai North Rd 229, Xi’an, Shaanxi Province 710069 People’s Republic of China; 2 Provincial Key Laboratory of Biotechnology of Shaanxi, Northwest University, Taibai North Rd 229, Xi’an, Shaanxi Province 710069 People’s Republic of China

**Keywords:** Parthenogenetic embryonic stem cells, Differentiation, Fibroblasts, Tissue-engineered skin equivalents

## Abstract

**Background:**

Embryonic stem cell (ESC) derivatives hold great promise for the construction of tissue-engineered skin equivalents (TESE). However, harvesting of ESCs destroys viable embryos and may lead to political and ethical concerns over their application. In the current study, we directed mouse parthenogenetic embryonic stem cells (pESCs) to differentiate into fibroblasts, constructed TESE, and evaluated its function in vivo.

**Methods:**

The stemness marker expression and the pluripotent differentiation ability of pESCs were tested. After embryoid body (EB) formation and adherence culture, mesenchymal stem cells (MSCs) were enriched and directed to differentiate into fibroblastic lineage. Characteristics of derived fibroblasts were assessed by quantitative real-time PCR and ELISA. Functional ability of the constructed TESE was tested by a mouse skin defects repair model.

**Results:**

Mouse pESCs expressed stemness marker and could form teratoma containing three germ layers. MSCs could be enriched from outgrowths of EBs and directed to differentiate into fibroblastic lineage. These cells express a high level of growth factors including FGF, EGF, VEGF, TGF, PDGF, and IGF1, similar to those of ESC-derived fibroblasts and mouse fibroblasts. Seeded into collagen gels, the fibroblasts derived from pESCs could form TESE. Mouse skin defects could be successfully repaired 15 days after transplantation of TESE constructed by fibroblasts derived from pESCs.

**Conclusions:**

pESCs could be induced to differentiate into fibroblastic lineage, which could be applied to the construction of TESE and skin defect repair. Particularly, pESC derivatives avoid the limitations of political and ethical concerns, and provide a promising source for regenerative medicine.

## Background

Tissue engineering is an efficient method to construct functional skin equivalents for the treatment of skin defects resulting from trauma or tumor excision [1]. The strategy involves the combination of keratinocytes or fibroblasts with certain cell seeding scaffolds. Cells seeded into the scaffolds secrete various growth factors and extracellular matrix (ECM) proteins, which could stimulate proliferation and differentiation of adjacent epithelial tissue, and accelerate wound healing. Prepare tissue-engineered skin equivalents (TESE) with autologous keratinocytes and fibroblasts would certainly be ideal. However, the process is time consuming, and several weeks are required to obtain the large cell numbers needed, which may put patients in danger of infection and dehydration. Furthermore, quality control of this customized therapeutic is difficult. Allogenic keratinocytes and fibroblasts for TESE could be harvested from foreskin circumcision specimens and cadaver skin. Despite the limited availability of the skin specimens for cell expansion, application of adult tissue has the risk of infectious pathogen transmission. Furthermore, these cells may lose their proliferative capacity and phenotype after extensive in-vitro passaging [2].

Embryonic stem cells (ESCs) have unlimited proliferative capacity and substantial ability to give rise to a variety of differentiated cell types [3]. In these situations, ESC derivatives are envisioned as promising candidates for tissue engineering and cell therapy [4, 5]. Great efforts have been made to establish protocols to manipulate ESCs to differentiate into specific phenotypes for the construction of desired tissue, such as bone, cartilage, and fat tissues [6–8]. Keratinocytes and fibroblasts derived from ESCs were also used successfully to construct TESE and stimulate skin defect healing [5, 9]. However, significant political and ethical concerns exist over the application of ESCs, because most ESCs are harvested from the inner cell mass of blastocysts and the process requires the destruction of viable embryos.

Parthenogenesis refers to the embryonic development of oocytes without fertilization. Uniparental parthenogenetic embryonic stem cells (pESCs) are a specific ESC type that could be obtained from the inner cell mass of blastocysts from chemically activated oocytes [10]. Because of difficulty in normal placenta formation, parthenogenetic embryos are unable to grow into viable fetuses in primates. This characteristic allows the application of pESCs and their derivatives while avoiding the ethical and political hurdles associated with biparental ESCs. pESCs possess typical characteristics similar to ESCs, such as extensive self-renewal ability and pluripotent differentiation capacity [11]. Importantly, uniparental pESCs are histocompatible because of their homozygosity in human leukocyte antigen (HLA) genotypes, which are more readily matched to patients and might reduce the risk of immune rejection after transplantation of their differentiated derivatives [12], thus offering significant advantages for the applications of cell-based therapies. Directed differentiation studies have shown that pESCs are capable of generating multiple cell lineages including cardiomyocytes, hepatocytes, pancreatic endocrine cells, retinal pigmented epithelial cells, and neural progenitor cells [11, 13–16]. Only one study has so far reported the application of pESC derivatives in stimulating damaged tissue repair [11].

We hypothesized that mouse pESCs could be directed to differentiate into fibroblasts for the construction of TESE. In the current experiment, after demonstrating that mouse pESCs exhibited similar fundamental properties to ESCs, we employed a stepwise approach to induce pESCs to differentiate into fibroblastic lineage. We then constructed TESE with pESC-derived fibroblasts (pFs) and evaluated the therapeutic effect with a mouse skin defects repair model.

## Methods

### Cell culture and characterization

The C57BL/6J pESC and J1 mouse ESC lines were cultured on dishes coated with 0.1 % (w/v) gelatin (Sigma-Aldrich, St. Louis, MO, USA) and expanded in Serum-Free Clonal Grade Medium (Millipore, Billerica, MA, USA). Cells were passaged every 5 days by 1 % accutase (Millipore), and observed by phase-contrast microscope (Nikon, Japan) during the process of culture.

Immunofluorescence staining was performed to detect stemness marker expression. pESCs and ESCs were plated on gelatin-coated (Sigma-Aldrich) glass cover lips and fixed by cold 4 % paraformaldehyde in phosphate-buffered saline (PBS) for 30 min, followed by washes three times with PBS and permeabilization with 0.25 % Triton X-100 (Sigma-Aldrich) for 10 min. Cells were then blocked with 10 % bovine serum albumin (BSA; Sigma-Aldrich) for 45 min and incubated overnight at 4 °C with 1:200 diluted primary antibodies, including goat anti-OCT3/4, rabbit anti-NANOG; and mouse anti-SSEA-1 (all from Santa Cruz Biotechnology, Santa Cruz, CA, USA). After three washes with PBS, the cells were incubated for 30 min at room temperature with fluorescein isothiocyanate (FITC)-labeled secondary antibodies (Invitrogen, Carlsbad, CA, USA). Nuclei were counterstained with 4,6′-diamidino-2-phenylindole (DAPI; Invitrogen). Images were obtained with a laser confocal microscope (FV1000; Olympus, Japan).

To confirm whether pESCs and ESCs possessed pluripotent differentiation capacity in vivo, ESCs and pESCs were dispersed using 1 % accutase, and 1 × 10^6^ cells were resuspended in 100 μl Dulbecco’s modified Eagle’s medium (DMEM; Gibco, Grand Island, NY, USA) and injected subcutaneously into nude mice. After 4 weeks, the specimens were harvested and fixed in 4 % paraformaldehyde, dehydrated through a graded ethanol, embedded in paraffin, sectioned at 7 μm, deparaffinized and stained with hematoxylin and eosin (H&E).

### Cell differentiation in embryoid bodies

pESCs and ESCs were dispersed and resuspended in DMEM supplemented with 20 % fetal bovine serum (FBS, Gibco), 50 U-μg/ml penicillin–streptomycin (Invitrogen). Subsequently, 1 × 10^5^ cells were transferred into ultra-low attachment dishes (Fisher Scientific, Pittsburgh, PA, USA) to form embryoid bodies (EBs). The medium was changed every 2 days. pESCs and ESCs were able to form EBs when cultured for 3 days, and were continuously cultured in suspension before surface antigen expression detection. EBs in suspension culture for 5 days were fixed in 4 % paraformaldehyde, dehydrated and embedded in paraffin, sectioned at 5 μm, and incubated with primary antibodies at 4 °C overnight. The primary antibodies comprised mouse anti-SSEA-1, rabbit anti-CD151 and cytokeratin (Santa Cruz Biotechnology), and rat anti-CD73 (eBioscience, San Diego, CA, USA). After removal of primary antibodies with three washes with PBS, FITC-labeled secondary antibodies (Invitrogen) were added and incubated for 1 hour at room temperature. The cells were washed three times with PBS and counterstained with DAPI, observed under a laser confocal microscope.

### Cell differentiation during adherent culture

EBs cultured in suspension for 5 days were plated onto 0.1 % (w/v) gelatin-coated dishes and cultured with DMEM supplemented with 20 % FBS, 50 U-μg/ml penicillin–streptomycin, 2 mM l-glutamine (Invitrogen), 1 % nonessential amino acids (Hyclone), 1 % β-mercaptoethanol (Sigma-Aldrich). To measure the expression of three germ layer markers, total RNA of adherently cultured EBs at different time points (5, 10, and 15 day) was isolated using TRIzol (Life Technologies, Carlsbad, CA, USA) according to the manufacturer’s instructions. Complementary DNA (cDNA) was synthesized from 1 μg of the normalized RNA samples using a RevertAid™ First Strand cDNA Synthesis Kit (Thermo Fisher Scientific, Waltham, MA, USA) following the manufacturer’s protocols. Relative levels of mRNA were determined from cDNA by quantitative real-time PCR with a SYBR Green PCR kit (Takara, Japan) in a total sample volume of 20 μl, and the samples were run in triplicate on a Bio-Rad CFX96 Real-Time PCR Detection System in accordance with the manufacturer’s instructions. The primer sequences and the fragment sizes are presented in Table 1. All primers were obtained from Takara. *Gapdh* was used as the reference gene. Single-peak melting profiles were obtained for the amplifications, and the size of the PCR product was confirmed by agarose gel electrophoresis. Each experiment was repeated three times. The ΔΔCT method [17] was used to calculate relative amounts of transcripts.

**Table 1:** Primers of three germ layer genes and the reference gene for quantitative real-time PCR

Gene	Primers (5′–3′)	Product (bp)
*Snai1*	GACCTGTGGAAAGGCCTTCTCTAGG	170
	CCTGGCACTGGTATCTCTTCACATC	
*Hand1*	GCTACGCACATCATCACCATCATC	125
	CAGCAGCCAGCTCTGGAAGTAAG	
*Gata2*	GCCAAAAGAGAGACTGGAGGAAGGG	82
	ACACCTCCCACCTTTTAGTCACTCTG	
nestin	GTTACCAAAGCCTCTTAGAAATGACC	577
	CAGATGCAACTCTGCCTTATCCTC	
*Oct3/4*	GTGTGAGGTGGAGTCTGGAG	182
	AGCCTCATACTCTTCTCGTTGG	
*Afp*	CTCTGGCGATGGGTGTTTAG	175
	TGCCTGGAGGTTTCGGGATT	
*Gapdh*	GGTGAAGGTCGGTGTGAACG	152
	CTCGCTCCTGGAAGATGGTG	

### Enrichment of MSCs from EB outgrowths

To enrich mesenchymal stem cells (MSCs), EBs cultured in suspension were adherently cultured as already described for 10–15 days. Cells were then cultured and expanded with MSC medium (MSCM; Lonza, Basel, Switzerland) for 5–6 passages to enrich spindle-shaped cells. Cells were passaged at a high ratio of 1:2 during expansion.

MSCs derived from pESCs and ESCs were named parthenogenetic MSCs (pMSCs) and embryonic MSCs (eMSCs), respectively. Cells were detached from culture dishes by Accutase, collected and washed three times with ice-cold PBS, and resuspended in PBS. FITC-conjugated primary antibodies (CD29, CD44, CD73; all from eBioscience) were added and incubated overnight, followed by two washes with ice-cold PBS. MSC surface antigen expressions were then tested by flow cytometry using FACS Calibur (BD Biosciences) analysis. Isotype-specific antibodies served as controls. Cells were analyzed using CellQuest software (BD Biosciences). At least 1 × 10^5^ cells were analyzed, and three independent tests were performed for each experiment.

For osteogenic differentiation, pMSCs and eMSCs were cultured in osteogenic differentiation medium (DMEM supplemented with 20 % FBS, 50 U-μg/ml penicillin–streptomycin, 50 μM ascorbic acid (Sigma-Aldrich), 10 mM β-glycerophosphate (Sigma-Aldrich), and 50 nM dexamethasone (Sigma-Aldrich)) for 21 days. The medium was changed every 3 days. After 21 days, cells were fixed in 4 % PBS-buffered paraformaldehyde and processed for Alizarin red S, Von Kossa staining, and reverse transcription-PCR (RT-PCR) assays for alkaline phosphates (*Alp*) and osteocalcin (*Ocn*), to test the osteogenic differentiation.

For chondrogenic differentiation, cells were cultured in chondrogenic medium (DMEM supplemented with 20 % FBS, 50 U-μg/ml penicillin–streptomycin, 50 μM ascorbic acid, 10 ng/ml transforming growth factor-β1 (TGF-β1; R&D Systems, Minneapolis, MN, USA), and 500 ng/ml insulin-like growth factor (IGF; R&D Systems)). The medium was changed every 3 days. After 21 days, cells were processed for Safranin O staining and PCR assays for aggrecan and type II collagen (*Col-II*).

For adipogenic differentiation, cells were exposed to adipogenic induction medium (DMEM supplemented with 20 % FBS, 50 U-μg/ml penicillin–streptomycin, 200 μM indomethacin (Sigma-Aldrich), and 10 μg/ml insulin (Sigma-Aldrich)) for 14 days. The medium was changed every 3 days. Adipogenic differentiation was indicated by oil-red O staining of lipid droplet formation in cytoplasm and RT-PCR assays for peroxisome proliferator-activated receptor gamma (*Pparγ*) and CCAAT/enhancer-binding protein alpha (*C/ebpα*).

For fibroblastic differentiation, cells were treated with DMEM supplemented with 20 % FBS, 50 U-μg/ml penicillin–streptomycin, 50 ng/ml recombinant human connective tissue growth factors (CTGF; BioVendor, Brno, Czech Republic) and 50 μM ascorbic acid, with medium changed every 3 days (pMSCs and eMSCs after CTGF induction were named pFs and ESC-derived fibroblasts (eFs), respectively). Growth factors in pFs and eFs were further measured every 5 days by enzyme-linked immunosorbent assays (ELISA) in triplicate. After the medium was removed by gentle aspiration using a vacuum manifold, the cells were then washed with PBS, and lysed with the addition of 1 ml RIPA buffer for total protein extraction. The cells were removed by scraping, and transferred into 1.5 ml conical tubes. The mixture was incubated on ice for 30 min, with occasional vortexing. The cell extracts were then assayed using mouse EGF, FGF, IGF1, VEGF, PDGFα, PDGFβ, TGFα, and TGFβ1 ELISA™ kits (Lian Shuo Biological, Shanghai, China) in accordance with the manufacturer’s instructions. Subsequently, the OD450 nm value was measured with an enzyme-labeled instrument (Thermo Fisher Scientific). The expression levels of mouse fibroblasts (Fs) were used as controls.

*Col-I*, *Col-III*, tenacin-C (*Tn-c*), matrix metalloproteinase-1 (*Mmp-1*), Vimentin, and fibroblast-specific protein-1 (*Fsp-1*) expression was screened by quantitative real-time PCR 20 days after induction. The primer sequences and the fragment sizes are presented in Table 2. To further detect the marker of derived fibroblasts, immunofluorescent staining was performed for rat anti-Vimentin (BOSTER), mouse anti-cytokeratin, goat anti-FSP1, and COL-I (all from Santa Cruz Biotechnology). Fibroblasts derived from pESCs and ESCs were named pFs and eFs, respectively.

**Table 2:** Primers of fibroblast phenotypic hallmark genes for quantitative real-time PCR

Gene	Primers (5′–3′)	Product (bp)
*Col-I*	TCTCCCCCAAGACACAGGAA	103
	GCTGGGTAGGGAAGTAGACG	
*Col-III*	ACTGTCCCACGTAAGCACTG	106
	CAGGAGGGCCATAGCTGAAC	
*Tn-c*	CCTACTGTCACGCGTCTCTC	112
	AAGCCACAACGAGTTCCCAA	
*Mmp1*	GAGCCACAGATGAGCACAGA	103
	AATCTGAACGCTCGCAGTGA	
vimentin	CGCTCCTACGATTCACAGCC	189
	TGTGGACGTGGTCACATAGC	
*Fsp1*	CTTCCTGTCCTGCATTGCCA	112
	GGCAAACTACACCCCAACAC	

### Preparation of TESE

To prepare TESE, 150 μl of 10 × DMEM was added into 1 ml ice-cold collagen solution (4 mg/ml type I collagen from rat tail dissolved in 0.1 % acetic acid). After neutralization with 500 μl 0.1 % NaOH solution, 100 μl of cell suspension (5 × 10^6^ cells/ml) was added into the solution and mixed immediately. The whole operation was carried out on ice. The mixture was transferred into dishes (*d* = 35 mm; Fisher Scientific, Pittsburgh, PA, USA) and then incubated at 37 °C and 5 % CO_2_ for gelling. Twenty-four hours later, 2 ml DMEM was added, and the TESE was cultured for another 2 days before use [18]. We prepared TESE from pFs, eFs, and Fs, and collagen gel without cells acted as control.

### TESE grafting onto mouse skin defects

Twenty BALB/c mice were purchased from the Experimental Animal Center of The Fourth Military Medical University and treated in accordance with the guidelines provided by the Institutional Ethics Committee of Northwest University. Mice were anesthetized by intraperitoneal injection of pentobarbital sodium (20 mg/kg body weight). Then 8 % sodium sulfate was used to depilate the wounding area of animals (instead of shaving) 24 hours before wounding, to ensure synchronization of hair growth. A circular full-thickness 1.5-cm-diameter skin defect was created on the back of each mouse using a biopsy punch (15 mm; purchased from Shanghai LZQ Precision Tool Technology Co., Ltd, Shanghai, China). TESE grafts derived from pFs, eFs, Fs, and control (*n* = 5 for each group) were implanted onto the skin defects. Grafts were covered with vaseline gauze and adhesive bandages for 3 days. Animal behavior and wounds were monitored throughout the experiment. Images were recorded at days 0, 3, 6, 9, 12, 15, and 18 post operation with a digital camera (Canon, Japan) to visualize the wound. The wound area was measured by tracing the wound margin and calculated using Image-Pro Plus Software (Media Cybernetics LP, Silver Spring, MD, USA). Investigators measuring samples were blind to groups and treatment. The wound closure percentage was calculated as follows:

$$ \left(\mathrm{Original}\ \mathrm{defect}\ \mathrm{area}\ \hbox{--}\ \mathrm{actual}\ \mathrm{defect}\ \mathrm{area}\right)\ /\ \mathrm{original}\ \mathrm{defect}\ \mathrm{area} \times 100\ \% $$

Animals were sacrificed at day 18, and the regenerated skin specimens (including dermis and epidermis) were fixed in 10 % buffered formalin for paraffin embedding. Sections were cut at 7 μm, deparaffinized, and stained with H&E. Sections were also stained with a mouse Cytokeratin-14 antibody (1:200; Santa Cruz Biotechnology), and a secondary Alexa-Fluor 594-labeled goat anti-mouse IgG (1:500; Invitrogen) was used.

### Statistical analysis

Data are expressed as mean ± SD of at least three independent samples. Statistical comparisons between groups were performed with one-way ANOVA and two-way ANOVA analysis. *p* < 0.05 and *p* < 0.01 were considered significant.

## Results

### pESCs exhibit properties similar to the pluripotent J1-ESC line

**Fig. 1:**
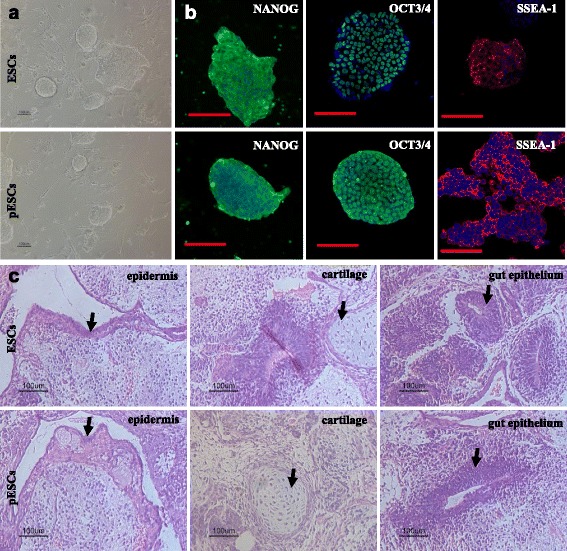
pESCs exhibit properties similar to the pluripotent J1-ESC line. **a** Morphological observations of pESCs and ESCs under phase-contrast microscopy. **b** Immunocytochemistry staining of embryonic stem cell markers for pESCs and ESCs. **c** H&E staining of teratoma sections from the pESCs and ESCs. The staining revealed that pESCs and ESCs possessed the capacity to generate epidermis, cartilage, and gut epithelium tissue in vivo. *Bars* = 100 μm. *ESC* embryonic stem cell, *pESC* parthenogenetic embryonic stem cell

ESCs and pESCs could form compact groups and proliferate actively (Fig. 1a). They expressed high levels of pluripotency gene markers, including NANOG, OCT3/4, and undifferentiated state marker SSEA-1 (Fig. 1b). Four weeks after injection of pESC and ESC suspension, tumor blocks could be observed on the back of the nude mice. Histological observation revealed that the newly formed teratomas contained three germ layers of epidermis (ectoderm), cartilage (mesoderm), and gut epithelium (endoderm) (Fig. 1c). These results demonstrated that pESCs exhibit similar fundamental properties to the well-characterized pluripotent J1-ESC line.

### pESCs are capable of differentiating into ectodermal, endodermal, and mesodermal cells in vitro

**Fig. 2:**
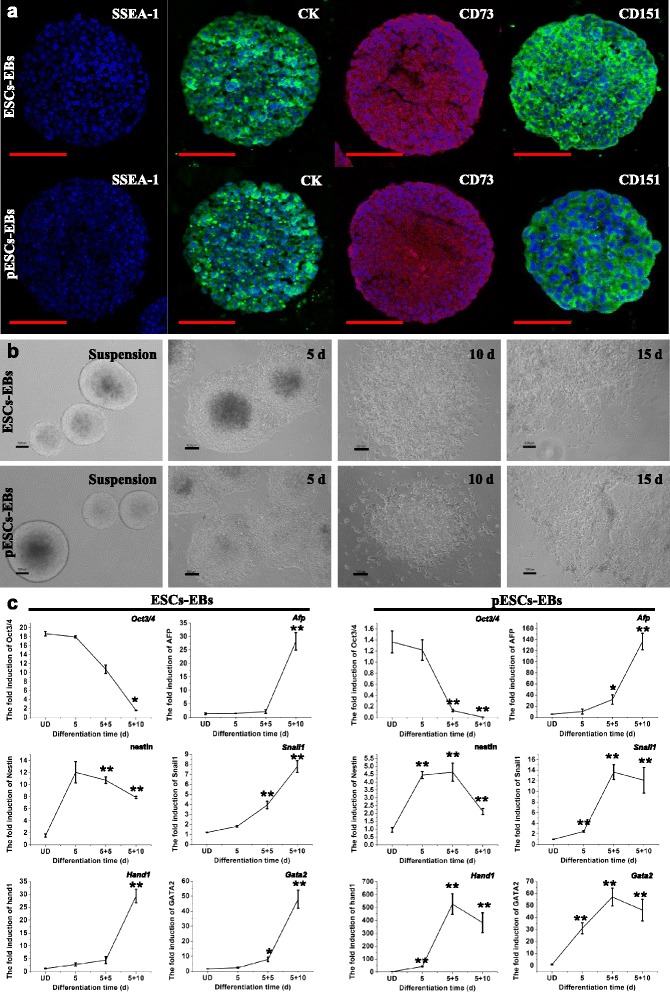
Characteristics of EB differentiation. **a** EB immunofluorescent staining of undifferentiated state marker SSEA-1 and surface antigens, including cytokeratin (*CK*), CD73, and CD151. Cell nuclei were stained with DAPI. *Bars* = 100 μm. **b** Morphological appearance of pESC-derived and ESC-derived EBs and outgrowths of EBs. *Bars* = 100 μm. **c** Expression of *Oct3/4* (stemness), nestin (ectoderm), *Afp* (entoderm), and mesoderm markers *Snai1*, *Hand1*, and *Gata2* in pESC-derived and ESC-derived EB outgrowths. *p < 0.05, **p < 0.01 vs undifferentiated (*UD*) cells. Each experiment was repeated three times. *EB* embryoid body, *ESC* embryonic stem cell, *pESC* parthenogenetic embryonic stem cell

pESCs and normal J1 ESCs formed EBs after 5 days of suspension culture. The immunofluorescent staining of EBs is shown in Fig. 2a. SSEA-1 expression was undetectable, demonstrating the initiation of differentiation. In addition, cytokeratin (ectoderm), CD73 (mesoderm), and CD151 (endoderm) expression became detectable. Gene expression profiles of EBs under adherent culture conditions revealed the programmed expression pattern related to the differentiation from ectodermal, mesodermal, and endodermal lineages, successively. The *Nestin* gene expression involved in ectoderm increased progressively 5 days after and declined 15 days after EB plating (Fig. 2c). The expression of mesodermal genes *Snail1*, *Hand1*, and *Gata2* also upregulated progressively, which peaked at 10 days of pESC-EB plating and at 15 days of ESC-EB plating (Fig. 2c). This was followed by expression of the endoderm-specific gene *Afp*, peaking at 15 days after plating. Pluripotent marker *Oct3/4* expression decreased rapidly at 5 days, and was almost undetectable at 15 days (Fig. 2c). The expression profile and level of genes in EBs of pESCs were similar to those of ESCs. Collectively, these data indicated that EBs from pESCs contained differentiated cells of all three germ layers and could be expanded during adherent culture.

### Enrichment and characterization of pMSCs

**Fig. 3:**
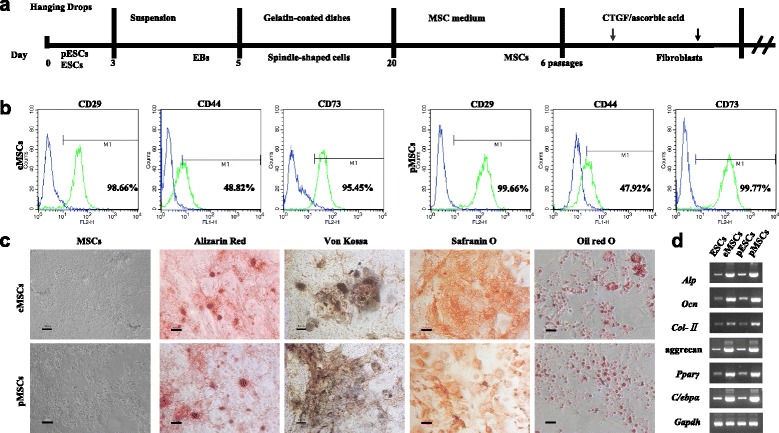
Multilineage differentiation and characteristics analysis of MSCs. **a** Schematic outline of pESC and ESC induction and fibroblastic differentiation flow chart. **b** Flow cytometry analysis of pMSCs and eMSCs indicates that these cells express MSC surface antigens. **c** Morphological appearance of pMSCs and eMSCs before osteogenic, chondrogenic, and adipogenic differentiation. Von Kossa and Alizarin Red S staining 21 days after osteogenic induction. Safranin O staining 21 days after chondrogenic induction. Oil Red O staining 14 days after adipogenic induction. *Bars* = 100 μm. **d** RT-PCR analysis of the gene expression profiles related to osteogenic, chondrogenic, and adipogenic differentiation. *EB* embryoid body, *eMSC* MSC derived from ESC, *ESC* embryonic stem cell, *MSC* mesenchymal stem cell, *pESC* parthenogenetic embryonic stem cell, *pMSC* MSC derived from pESC

Spindle-shaped cells could migrate from EBs 8–10 days after plating. When cultured in MSC medium in high density for 5–6 passages, cells derived from EBs exhibited fibroblastic morphology (Fig. 2b). The formation of MSCs is the intermediate stage during the process of ESC differentiation. Hence, we enriched eMSCs and pMSCs, which could be subcultured for up to 10 passages without obvious morphological changes. FACS analysis confirmed the positive expression of CD29, CD44, and CD73, particularly, with more than 95 % of cells expressing CD73 (mesodermal marker) (Fig. 3b).

MSCs are functionally characterized by their ability to differentiate into mesenchymal tissues including bone, cartilage, and fat. Therefore, we tested whether pMSCs and eMSCs have the same potential. Cells of passage 5 were subjected to osteogenic, chondrogenic, and adipogenic differentiation in vitro using the standard protocol to confirm multilineage differentiation capability. Strong staining for Von Kossa and Alizarin Red staining 21 days after induction demonstrated calcium deposition in the matrix (Fig. 3c), together with upregulation of *Alp* and *Ocn* expression, indicating that pMSCs and eMSCs had osteogenic potential (Fig. 3d).

After chondrogenetic induction by TGF-β1 and IGF for 21 days, strong staining for Safranin O could be observed around cells, indicating specific ECM of proteoglycan secretion and deposition. Typical mirror image cells were embedded in the matrix, verifying the chondrogenic lineage differentiation of these cells (Fig. 3c). Chondrogenic differentiation was also confirmed by the unregulated gene expression of *Col-II* and aggrecan, two components of ECM selectively expressed by chondrocytes, using RT-PCR (Fig. 3d).

Adipocytic differentiation of pMSCs and eMSCs was induced under conditions described previously. Appearance of cells harboring fat granules could be observed after induction for 14 days in culture, which is positive for Oil Red O staining (Fig. 3c). At the same time, increased expression of *Pparγ* and *C/ebpα*, markers of adipocytic differentiation, was found upregulated during the process (Fig. 3d). Collectively, these results demonstrated that pMSCs and eMSCs have the multilineage differentiation capacity, and could be directed to differentiate into bone, cartilage, and fat.

### Fibroblastic differentiation of pMSCs

**Fig. 4:**
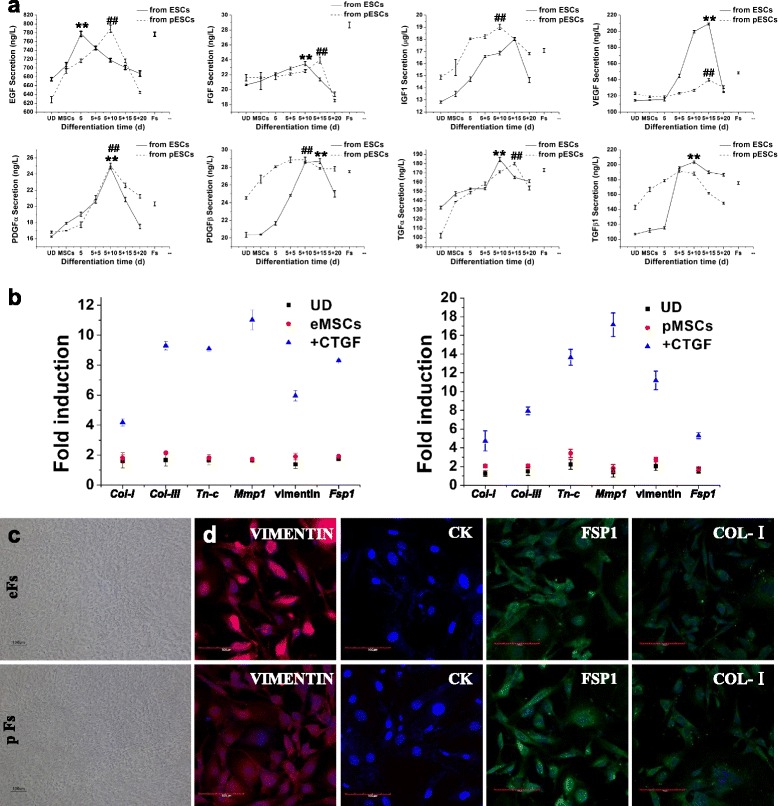
Fibroblastic differentiation of MSCs and growth factor expression evaluation. **a** Protein expression levels of growth factors in induced and undifferentiated (*UD*) pESCs and ESCs by ELISA. **p < 0.01 and ^##^ *p* < 0.01 vs Fs. Each experiment was repeated three times. **b** Quantitative RT-PCR assay of fibroblast phenotypic hallmarks including *Col-I, Col-III, Tn-c, Mmp1,* vimentin, and *Fsp1* expression 20 days after fibroblastic differentiation. **c** Morphological appearance of fibroblasts derived from pMSCs and eMSCs after CTGF induction under microscopy analysis. *Bars* = 100 μm. **d** Immunofluorescent staining of fibroblasts derived from pMSCs and eMSCs for VIMENTIN, CYTOKERATIN (*CK*), FSP1, and COL-I. Cell nuclei were stained with DAPI. *Bars* = 100 *μ*m. *eF* ESC-derived fibroblast, *eMSC* MSC derived from ESC, *ESC* embryonic stem cell, *MSC* mesenchymal stem cell, *pESC* parthenogenetic embryonic stem cell, *pF* pESC-derived fibroblast, *pMSC* MSC derived from pESC

As paracrine of growth factors is critical to skin regeneration, we compared the secretory profile of eFs, pFs, and Fs for selected growth factors. ELISA results revealed that pFs and eFs expressed similar elevated levels of growth factors including EGF, FGF, IGF1, TGFα, TGFβ1, PDGFα, and PDGFβ after induction with CTGF. Both pFs and eFs expressed a higher level of PDGFα (15 days after induction) and a lower level of FGF than Fs. Notably, the expression of EGF in eFs was significantly higher than that in pFs and Fs (15 and 20 days after induction) (Fig. 4a).

Quantitative real-time PCR detection indicated that after 20 days of treatment with 50 ng/ml CTGF, the expression of fibroblast phenotypic hallmarks including *Col-I, Col-III, Tn-c, Mmp1,* vimentin, and *Fsp1* increased drastically in pFs and eFs (Fig. 4b). Morphological appearances of fibroblasts derived from pMSCs and eMSCs are shown in Fig. 4c. Immunofluorescent staining further demonstrated that these cell were positive for VIMENTIN, FSP1, and COL-I, but negative for CK (Fig. 4d).

### TESE from pFs promote mouse wound healing in vivo

**Fig. 5:**
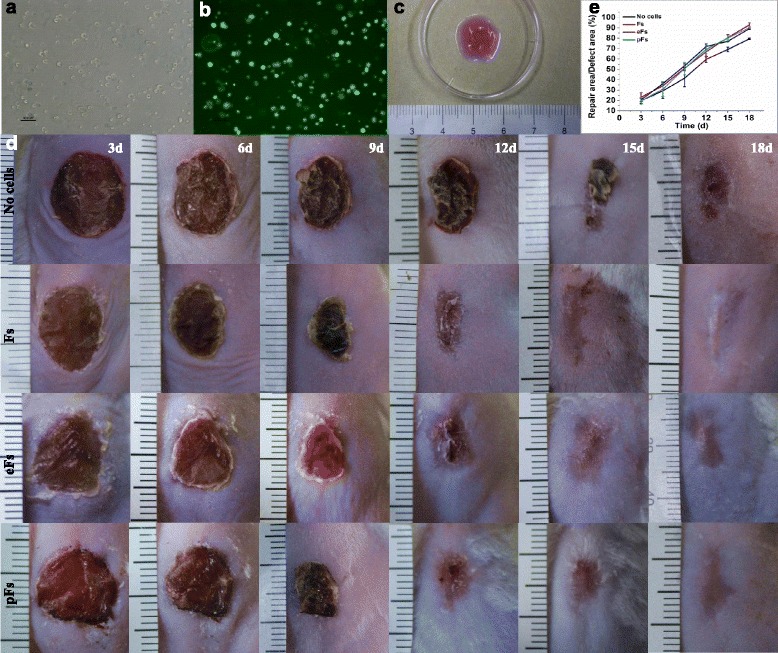
Preparation of TESE and grafting onto mice. **a** Homogeneous cell encapsulation in the collagen gels. *Bars* = 100 μm. **b** CFDA labeling shows the high viability of embedded cells in the collagen gels. *Bars* = 100 μm. **c** Gross appearance of prepared TESE before use. **d** Gross appearance of skin defect repair. **e** Mean wound closure percentage for each group in a time manner. Error bars represent SDs, *n* = 5 for each group. Statistical results are presented in Table 4. *eF* embryonic stem cell-derived fibroblast, *F* mouse fibroblast, *pF* parthenogenetic embryonic stem cell-derived fibroblast

Gross appearance of TESE prepared from pFs is shown in Fig. 5c. Phase-contrast microscope observation indicated that cells were homogeneously distributed in the collagen gels and displayed a rounded morphology (Fig. 5a). Viable cells stained green could be noticed after CFDA labeling (Fig. 5b), indicating TESE could be generated from pFs and eFs.

**Fig. 6:**
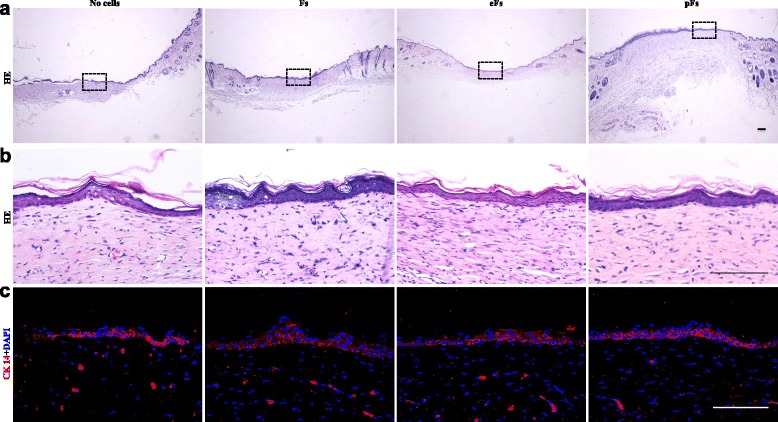
H&E and immunofluorescent staining of repaired skin tissues 18 days after TESE grafting onto mouse skin defects. **a** Low-power and **b** high-power magnificent H&E staining of repaired skin tissues 18 days after grafting. *Bars* = 100 μm. **c** Immunofluorescent staining of repaired skin tissues 18 days after grafting for cytokeratin-14 (*CK 14*). Cell nuclei were stained with DAPI. *Bars* = 100 μm. *eF* embryonic stem cell-derived fibroblast, *F* mouse fibroblast, *pF* parthenogenetic embryonic stem cell-derived fibroblast

All of the animals survived and no visible inflammation occurred during the process of wound healing. Gross inspections showed an increasing reduction in the area of the defects in all groups during the experiment and were digitally recorded on days 0, 3, 6, 9, 12, 15, and 18 after TESE grafting (Fig. 5d). As shown in Fig. 5e, the wound repair rate after different TESE grafting was quantitatively measured. The collagen gel without cells showed the lowest capacity for the repair of the skin defect and by day 15 the overall wound repair rate was 69.1 %, which was significantly lower compared with the other groups (*p* < 0.05), and the skin defects were re-epithelialized by day 18. The F-TESE group exhibited the highest wound closure rate and most of the skin defect was repaired by day 15 (80.5 % wound closure rate). Similar to the F-TESE group, wound closure rates were 80.1 % and 77.0 % in the pF-TESE and eF-TESE group, respectively, and there was no significant difference compared with the F-TESE group (*p* > 0.05) (Tables 3 and 4). Skin specimens was harvested at day 18 and processed for histological examination to detect the quality of the newly repaired tissue. Cytokeratin-14 immunostaining showed that cytokeratins existed in all groups, indicating that the skin defects have been re-epithelialized 18 days post operation. Three cell seeding groups (pFs, eFs, and Fs seeded into collagen gel) showed better skin structure and stronger CK-14 staining (Fig. 6).

**Table 3:** Wound repair rate (%) after different TESE grafting in a time manner

	3 days	6 days	9 days	12 days	15 days	18 days
No cell	19.77 ± 2.46	29.31 ± 7.46	41.34 ± 8.22	59.57 ± 3.10	69.05 ± 2.26	79.38 ± 0.76
Fibroblasts	23.14 ± 4.28	34.09 ± 3.81	50.87 ± 2.34	66.85 ± 4.98	80.5 ± 3.10	92.92 ± 1.96
eMSCs	20.71 ± 1.66	36.12 ± 1.02	53.32 ± 1.98	71.91 ± 2.63	77.01 ± 0.52	89.20 ± 0.71
pMSCs	19.70 ± 3.23	30.86 ± 7.14	50.59 ± 6.03	69.25 ± 4.12	80.06 ± 2.74	90.20 ± 0.78

Data presented as mean ± SD

*eMSC* mesenchymal stem cell derived from embryonic stem cell, *pMSC* mesenchymal stem cell derived from parthenogenetic embryonic stem cell, *TESE* tissue-engineered skin equivalents

**Table 4:** Statistics analysis of wound repair rate between groups 15 days after different TESE transplantation

	No cells	Fs	eFs
Fs	*p* < 0.05		
eFs	*p* < 0.05	*p* > 0.05	
pFs	*p* < 0.05	*p* > 0.05	*p* > 0.05

*F* mouse fibroblast, *eF* embryonic stem cell-derived fibroblast, *pF* parthenogenetic embryonic stem cell-derived fibroblast, *TESE* tissue-engineered skin equivalents

## Discussion

Recent progress in regenerative medicine and tissue engineering has focused on the application of ESC derivatives to improve regeneration of targeted tissues [19, 20], due to their unlimited proliferative capacity and the potential to give rise to a variety of differentiated cell types. Several studies have so far reported the successful preparation of skin equivalents using keratinocytes or fibroblasts derived from ESCs. However, political and ethical limitations may hinder the application of ESCs and their derivatives. Induced pluripotent stem (iPS) cells obtained by reprogramming of somatic cell have also drawn much attention [21], and directed differentiation of iPS cells into >several cell lineages have also been reported in detail [22, 23]. But the major limitations for iPS cells is timely allocation (usually within weeks to several months), which make them inapplicable for the treatment of acute disease conditions including large burns, heart failure, bone, and cartilage injuries.

pESCs are derived from the blastocysts of chemically activated and subsequently developed oocytes. Because parthenogenetic blastocysts are not able to develop into an embryo, the ethical concerns relating to the application of ESCs derived from fertilized oocytes could be avoided [10]. Importantly, haploid identity of major histocompatibility complex (MHC) of pESCs may increase immune tolerance after allogeneic cell transplantation and decrease the number of cell lines needed for therapeutic cell banking [24–26]. One of the main challenges in using pESCs for regenerative medicine is the difficulty of directing pESC differentiation into the interested phenotype. In a pioneer study, Michel et al. induced pESC differentiation toward the cardiac lineage and tested its application in tissue-engineered heart repair [11]. The results showed that cardiomyocytes could be obtained to facilitate engineering of force-generating myocardium and demonstrated the utility of this technique in enhancing regional myocardial function after myocardial infarction. Importantly, their results also demonstrated the immunological acceptance of pESC allografts in related and unrelated recipients with matched MHC. We have successfully fabricated injectable adipose tissue with pESC-derived adipocytes [27].

In the current study, we aimed to determine whether pESCs could be directed to differentiate into fibroblasts in vitro, and to evaluate the application in tissue-engineered skin defect repair. We first demonstrated that pESCs and ESCs had similar fundamental differentiation potential. The teratoma formation test demonstrated that both pESCs and ESCs could differentiate into epidermis (ectoderm), cartilage (mesoderm), and gut epithelium (endoderm) in vivo. Importantly, hyaline cartilage could be observed in the teratoma tissue after pESC injection, indicating that pESCs have strong potential to differentiate into mature mesenchymal tissue (Fig. 1). PCR and immunofluorescent observation demonstrated that cells in EBs could spontaneously differentiate into cells of three germ layers, as indicated by gene expression of nestin (ectoderm), *Afp* (endoderm), and *Snail1*, *Hand1*, and *Gata2* (mesoderm) (Fig. 2). Interestingly, we found pESC-EB outgrowth underwent earlier differentiation compared with ESC-EB. As indicated by RT-PCR assay, the expression of mesodermal genes from pESC-EB outgrowth peaked at 10 days after plating, while expression peaked at 15 days after ESC-EB plating (Fig. 2c). In accordance with this result, the expression of *Oct3/4* in pESC-EB outgrowth was much lower than that of ESCs-EBs at 10 days of plating.

We then directed pESCs to differentiate into fibroblasts with stepwise induction. Adherent culture is critical to obtain targeted cell numbers for application. After adherent culture of EBs for 10 or 15 days, MSC culture medium was applied and cells were subcultured for 5–6 passages in high density to enrich MSCs from EB derivatives. Flow cytometry detection indicated that pMSCs and eMSCs expressed MSC markers (Fig. 3). Multipotent differentiation investigation showed that the expanded cell populations could be induced to differentiate into osteogenic, chondrogenic, and adipogenic lineages (Fig. 3c, d), respectively, which were in agreement with the results from Hwang et al. [28].

It has been reported that adult MSCs can also differentiate into fibroblastic lineage under the induction of CTGF [29]. We next tested whether pMSCs and eMSCs possess this potential under the conditions described previously. After treatment with CTGF, fibroblast phenotypic hallmarks drastically increased, including *Col-I , Col-III, Tn-c, Mmp1,* vimentin, *and Fsp1* (Fig. 4b, c). Importantly, these cells also expressed a high level of growth factors after induction, which is critical for skin defect repair and wound healing. Compared with normal fibroblasts, pFs exhibited a similar or higher expression level of EGF, IGF1, VEGF, TGFα, TGFβ1, PDGFβ, and PDGFα (Fig. 4a).

Finally we tested pF-derived and eF-derived TESE for the repair of skin defects. In accordance with our hypothesis, pFs were similar to Fs and eFs not only phenotypically but also in functional terms. In-vivo experiments indicated that pF-derived TESE are as efficient as normal fibroblasts and eF-derived TESE for the re-epidermalization of the skin defects (Fig. 5d, e). More than 80 % of skin defects (80.06 ± 2.74) were repaired 15 days after pF-derived TESE transplantation (Table 3).

## Conclusions

One of our key findings in the current study is that pESCs could be directed into a fibroblastic lineage via the middle stage of MSCs, facilitating for tissue-engineered skin defect repair. pMSCs have strong potential to differentiate into osteogenic and chondrogenic lineages, suggesting that pMSCs are also facilitated for bone and cartilage tissue engineering. Compared with ESCs and iPS cells, pESCs have ethical, immunological, and technical (no genetic manipulation required) advantages. Collectively, these characteristics, together with the ease and high efficiency of directed differentiation, warrant pESCs as an optimizing source for tissue engineering and regenerative medicine.

## Abbreviations

ALP: Alkaline phosphates
C/EBPα: CCAAT/enhancer-binding protein alpha
EB: Embryoid body
ECM: Extracellular matrix
eMSCs: MSCs derived from ESCs
ESC: Embryonic stem cell
FSP1: Fibroblast-specific protein-1
MMP-1: Matrix metalloproteinase-1
MSC: Mesenchymal stem cell
OCN: Osteocalcin
pESC: Parthenogenetic embryonic stem cell
pMSCs: MSCs derived from pESCs
PPARγ: Peroxisome proliferator-activated receptor gamma
TESE: Tissue-engineered skin equivalents
